# Statement of Retraction: Cinnamaldehyde attenuates kidney senescence and injury through PI3K/Akt pathway-mediated autophagy via downregulating miR-155

**DOI:** 10.1080/0886022X.2026.2740219

**Published:** 2026-09-30

We, the Editors and Publisher of *Renal Failure*, have retracted the following article:

Xiao, Q. (2022). Cinnamaldehyde attenuates kidney senescence and injury through PI3K/Akt pathway-mediated autophagy via downregulating miR-155. *Renal Failure*, *44*(1), 601–614. https://doi.org/10.1080/0886022X.2022.2056485.

Following publication, the journal was contacted by the author in 2025 to request withdrawal of the published article as they had identified additional data which they would like to publish.

Following this request, investigation by the Publisher identified the below concerns:

- Image integrity concerns regarding staining Figures 2B D-Gal + VE (X100) and 2B D-Gal + VE (X400)
- Image integrity concerns regarding Western Blot Figures 1D, 2E, 3D
- qRT-PCR methods and results described in the study.

When approached for an explanation, the authors did not respond to our queries. As verifying the ­validity of published work is core to the integrity of the scholarly record, we are therefore retracting the article. The corresponding author has been informed.

We have been informed in our decision-making by our editorial policies and the COPE guidelines.

The retracted article will remain online to maintain the scholarly record, but it will be digitally ­watermarked on each page as ‘Retracted’.

